# An observational study to identify causative factors for not using hydroxychloroquine in systemic lupus erythematosus

**DOI:** 10.1038/s41598-024-58463-3

**Published:** 2024-04-02

**Authors:** Atsushi Manabe, Ryuichi Minoda Sada, Hirofumi Miyake, Hiroyuki Akebo, Yukio Tsugihashi, Kazuhiro Hatta

**Affiliations:** 1 Department of General Internal Medicine, Tenri Hospital, Tenri, Japan; 2 Department of Rheumatology and Clinical Immunology, Graduate School of Medicine, Kyoto University, Kyoto, Japan; 3 Department of Infection Control, Graduate School of Medicine, Osaka University, Suita, Japan; 4 Department of Transformative Protection to Infectious Disease, Graduate School of Medicine, Osaka University, 2-2 Yamadaoka, Suita, Osaka 565-0871 Japan; 5 Medical Home Care Centre, Tenri Hospital, Shirakawa Branch, Tenri, Japan

**Keywords:** Hydroxychloroquine, Non-use, Factor, Systemic lupus erythematosus, Systemic lupus erythematosus, Systemic lupus erythematosus

## Abstract

Hydroxychloroquine (HCQ) use is indicated for patients with systemic lupus erythematosus (SLE). Nevertheless, reports discussing the reasons for not prescribing HCQ are limited. We identified the factors that interfere with HCQ use in patients with SLE. This observational, single-center study included data from 265 patients with SLE in 2019. The patients were categorized into groups with and without a history of HCQ use. Between these groups, clinical characteristics were compared using univariate analysis and logistic regression models. Among the 265 patients, 133 (50.2%) had a history of HCQ use. Univariate analysis identified older age; longer disease duration; lower prednisolone dose, clinical SLE disease activity index 2000, and estimated glomerular filtration rate; higher C3 level; and lower anti-double-stranded DNA antibody concentration as HCQ non-use-related variables. Logistic regression models identified a positive association between HCQ non-use and longer disease duration (odds ratio [OR] 1.08), prednisolone dose ≤ 7.5 mg/day (OR 4.03), C3 level ≥ 73 mg/dL (OR 2.15), and attending physician having graduated > 10 years prior (OR 3.19). In conclusion, a longer disease duration, lower prednisolone dose, higher C3 level, and longer time since attending physicians’ graduation correlated with HCQ non-use. Physicians and patients should be educated to facilitate HCQ use despite these factors.

## Introduction

Hydroxychloroquine (HCQ) is recommended for all patients with systemic lupus erythematosus (SLE) unless contraindicated^1–3^. HCQ has several beneficial effects in patients with SLE, including a reduced risk of clinical flares^4^ and organ damage^5^, and improves overall survival^6,7^. Particularly, it improves skin diseases^8^ and arthritis^9^, among various organ lesions. Additionally, HCQ reduces the risk of infection^10^ and has antithrombotic^11^, antidiabetic^12^, and lipid-lowering effects^13^.

In Japan, HCQ was unavailable until 2015 because of the ban on chloroquine in 1974 following a series of retinopathy complications^14^. The literature indicates a low frequency of HCQ use (18.3%), as shown in the lupus registry of nationwide institutions (LUNA), which is one of the largest multicenter cohorts in Japan^15^. However, only a few reports are available regarding the reasons for not prescribing HCQ.

Therefore, this study aimed to identify the percentage of patients with SLE using HCQ and the factors for not prescribing HCQ in these patients.

## Materials and methods

### Study design and settings

This observational, single-center study used data from the Department of General Internal Medicine at Tenri Hospital on October 31, 2019. Tenri Hospital is a regional tertiary care teaching hospital in Japan with 715 beds. Medical care for rheumatic diseases is provided by the Department of General Internal Medicine, which is composed of 13 physicians, including 3 rheumatologists. Rheumatologists were defined as holders of qualifications certified by the Japan College of Rheumatology, regardless of whether they had other licenses of medical specialists, including general medicine. The attending physician graduated with a median (interquartile ranges [IQR]) of 6 (4–8) years previously with a maximum and minimum of 37 and 3 years, respectively. At Tenri Hospital, patients can visit the ophthalmology department without difficulty for baseline examination before HCQ use and annual screening for retinopathy, which is a severe side effect of HCQ. Clinical characteristics were compared between patients with and without HCQ use. This study followed the Strengthening the Reporting of Observational Studies in Epidemiology (STROBE) statement for observational studies. This study protocol was approved by the Institutional Review Board of Tenri Hospital (Number 1212) and was conducted in accordance with the tenets of the Declaration of Helsinki. An opt-out method was used to provide explanations to the patients and obtain consent.

### Study participants

All adult patients with SLE based on the 1997 update by the 1982 American College of Rheumatology (ACR)^16^ who continued visiting our outpatient clinic until the end of October 2019 were included in this study. The history of HCQ in the study population was investigated through electronic medical records. The patients were classified into two groups as follows: one without any history of HCQ use (non-HCQ group) and the other with a history of HCQ use (HCQ group). Additionally, the proportion of patients who belonged to the HCQ group was calculated.

### Variables and data source

Variables included sex, age, disease duration, clinical manifestations, clinical SLE disease activity index 2000 (clinical SLEDAI-2K)^17,18^, which is a measure of lupus activity, therapeutic agents (prednisolone [PSL] dose and the use of immunosuppressive drugs [azathioprine, tacrolimus, cyclosporine, mycophenolate mofetil, methotrexate, and mizoribine] and belimumab), and laboratory data (estimated glomerular filtration rate [eGFR] and level of aspartate aminotransferase [AST], complement represented by C3, and anti-double-stranded DNA antibodies [anti-dsDNAs]). AST was included because of the concern about the increase in adverse events in patients with hepatic dysfunction, which is supported by the relationship between Cytochrome P450 enzymes and blood concentrations of HCQ^19,20^. Continuous variables were entered after dichotomization into categorical variables using clinically significant values, where PSL dose was separated at 7.5 mg according to the Lupus Low Disease Activity State criteria^21^. The proportion of attending physicians as of 2019 with ˃ 10 years of experience since graduation was also investigated. The cut-off for years since graduation was set at 10 because it is the approximate number of years required to obtain a rheumatologist’s license in Japan. Even in cases with a history of changes in attending physicians, the attending physician, as of 2019, was treated as the representative. Aside from years since graduation of the attending physicians, data from the last visit in the study period in the non-HCQ group and the date of first use of HCQ in the HCQ group was obtained. The date in the HCQ group was determined based on the significant clinical information at the time HCQ was supposed to be initiated.

### Statistical analysis

Continuous and categorical variables are expressed as medians with IQR and the number of patients with percentages, respectively. Differences between the two groups were analyzed using the Mann–Whitney U and Fisher exact tests for continuous and categorical variables, respectively. Multivariate analysis was used to identify parameters that were independently associated with HCQ non-use. A binary logistic regression model was fitted with HCQ non-use as the dependent variable. Aside from age and sex, the characteristics that were significantly different in univariate analyses were entered as independent variables. Therefore, to assess the presence of collinearity, the variance inflation factor was calculated and conservatively classified values of ≥ 10 as suggestive of collinearity. The goodness-of-fit of the final model was tested by calculating the area under the receiver operating characteristic curve. Additionally, a sensitivity analysis was performed where the categorical variables were changed to continuous variables. Finally, the subgroup diagnosed with SLE before 2015 was analyzed because which of the diagnosis of SLE and the approval of HCQ preceded the other could have been a potential confounder. Data points with missing values were removed from each univariate and multivariate analysis. For all analyses, statistical significance was considered at p ˂ 0.05. All statistical analysis was performed using IBM SPSS Statistics for Windows version 22 (IBM Corp., Armonk, NY, USA).

## Results

Overall, 469 patients were enrolled, and 265 were eligible. All patients were Asian, and 241 (90.9%) were female. The median (IQR) age was 50 (40–66) years, disease duration was 11 (4–23) years, clinical SLEDAI-2K was 3 (1–5), and PSL dose was 5 (3–10) mg/day. The proportion of HCQ users was 50.2% (132/265).

The clinical features compared between the non-HCQ and HCQ groups and the results of the univariate analysis are shown in Table 1. Patients in the non-HCQ group were older, had a longer disease duration, and received a lower PSL dose. The PSL dose, clinical SLEDAI-2K, eGFR, and anti-dsDNA expression were lower, and C3 level was higher in the non-HCQ group. Additionally, the proportion of attending physicians with experience of ˃ 10 years since graduation was higher in the non-HCQ group than in the HCQ group.

**Table 1:** Univariate analysis for factors associated with non-use of hydroxychloroquine in patients with systemic lupus erythematosus.

Variable	Non-HCQ group (n = 132)	HCQ group (n = 133)	p-value
Sex (female), n/N (%)	121/132 (91.7)	120/133 (90.2)	0.83
Age (years)	57 (46–69)	45 (35–59)	< 0.001
Disease duration (years)	20 (12–28)	5 (2–11)	< 0.001
Clinical manifestations, n/N (%)			
Malar rash	67/132 (50.8)	59/133 (44.4)	0.33
Discoid rash	42/132 (31.8)	33/133 (24.8)	0.22
Photosensitivity	56/132 (42.4)	51/133 (38.3)	0.53
Oral ulcers	23/132 (17.4)	15/133 (11.3)	0.16
Arthritis	78/132 (59.1)	77/133 (57.9)	0.90
Serositis	24/132 (18.2)	35/133 (26.3)	0.14
Renal disorder	51/132 (38.6)	43/133 (32.3)	0.31
Neurological disorder	17/132 (12.9)	12/133 (9.0)	0.33
Hematologic disorder	116/132 (87.8)	110/133 (82.7)	0.30
Clinical SLEDAI-2K = 0, n/N (%)	35/132 (26.5)	11/132 (8.3)	< 0.001
PSL ≤ 7.5 mg/day, n/N (%)	114/132 (86.4)	72/133 (54.1)	< 0.001
Immunosuppressive drugs, n/N (%)	80/132 (60.6)	79/133 (59.4)	0.90
AZA, n/N (%)	8/132 (6.1)	7/133 (5.3)	0.80
TAC, n/N (%)	14/132 (10.6)	14/133 (10.5)	1.0
CyA, n/N (%)	17/132 (12.9)	15/133 (11.3)	0.71
MMF, n/N (%)	9/132 (6.8)	10/133 (7.5)	1.0
MTX, n/N (%)	2/132 (1.5)	5/133 (3.8)	0.45
MZR, n/N (%)	6/132 (4.5)	7/133 (5.3)	1.0
BLM, n/N (%)	1/132 (0.8)	0/133 (0)	0.50
eGFR < 60 mL/min/1.73 m^2^, n/N (%)	37/131 (28.2)	17/133 (12.8)	0.002
AST > 30 IU/L	23/131 (17.6)	26/133 (19.5)	0.75
C3 ≥ 73 mg/dL, n/N (%)	96/128 (75.0)	69/133 (51.9)	< 0.001
Anti-dsDNA ≤ 12 IU/mL, n/N (%)	75/122 (61.5)	52/119 (43.7)	0.007
Attending physicians with > 10 years since graduation, n/N (%)	107/132 (81.1)	58/133 (43.6)	< 0.001

Data presented as median (interquartile range) of patients unless otherwise indicated. In the analysis of disease duration, the non-HCQ and HCQ group consisted of 127 and 130 patients. In columns related to C3 and dsDNA, the cut off values represent the lower and upper limits of normal, respectively.

*HCQ* hydroxychloroquine, *Clinical SLEDAI-2K* clinical systemic lupus erythematosus disease activity index 2000, *PSL* prednisolone, *eGFR* estimated glomerular filtration rate, *AST* aspartate aminotransferase, *AZA* azathioprine, *TAC* tacrolimus, *CyA* cyclosporine, *MMF* mycophenolate mofetil, *MTX* methotrexate, *MZR* mizoribine, *BLM* belimumab.

The results of the multivariate analysis are presented in Table 2. HCQ non-use was positively correlated with longer disease duration (years) (odds ratio [OR] 1.08, 95% confidence interval [CI] 1.04–1.12, p < 0.001), PSL dose ≤ 7.5 mg/day (OR 4.03, 95% CI 1.80–9.01, p = 0.001), C3 level ≥ 73 mg/dL (OR 2.15, 95% CI 1.01–4.56, p = 0.046), and attending physicians’ years since graduation > 10 years (OR 3.19, 95% CI 1.54–6.61, p = 0.002). Collinearity diagnostics did not identify any variables with a variance inflation factor of ≥ 10. The area under the receiver operating characteristic curve was 0.86 (95% CI 0.82–0.91). These findings were approximately consistent with the sensitivity analyses where the categorical variables were changed to continuous variables (Supplementary Tables S1, S2). Similarly, the subgroup analysis for the patients diagnosed before 2015 showed consistent results (Supplementary Tables S3, S4).

**Table 2:** Multivariate analysis using logistic regression model for factors associated with non-use of hydroxychloroquine in patients with systemic lupus erythematosus.

Variable	Odds ratio (95% CI)	p-value
Female sex	0.61 (0.17–2.10)	0.43
Age, per year	1.00 (0.98–1.03)	0.87
Disease duration, per year	1.08 (1.04–1.12)	< 0.001
Clinical SLEDAI-2K ≤ 4	2.25 (0.75–6.74)	0.15
PSL ≤ 7.5 mg/day	4.03 (1.80–9.01)	0.001
eGFR < 60 mL/min/1.73 m^2^	1.78 (0.73–4.36)	0.21
C3 ≥ 73 mg/dL	2.15 (1.01–4.56)	0.046
Anti-dsDNA ≤ 12 IU/mL	1.04 (0.51–2.11)	0.92
Attending physicians with > 10 years since graduation	3.19 (1.54–6.61)	0.002

The logistic regression model analyzed non-use of hydroxychloroquine as the dependent variable against column variables as independent variables, including participants with no missing values (n = 233). In columns related to C3 and dsDNA, the cut off values represent the lower and upper limits of normal, respectively.

*CI* confidence interval, *Clinical SLEDAI-2K* clinical systemic lupus erythematosus disease activity index 2000, *PSL* prednisolone, *eGFR* glomerular filtration rate.

## Discussion

In this observational study, the proportion of HCQ use was 50.2%, and longer disease duration, lower PSL dose, higher C3 level, and more years since graduation of attending physicians were associated with HCQ non-use. However, whether HCQ is prescribed inappropriately because of these factors remains a concern. Therefore, HCQ use should be considered even with these factors, considering the recommendation of HCQ for SLE^1–3^ and several beneficial effects, including a protective effect on overall survival^4–13^.

In this study, a lower PSL dose, longer disease duration, higher C3 level, and attending physicians’ years since graduation were significantly associated with HCQ non-use. The following are the underlying reasons for these associations. Lower PSL dose, longer disease duration, and higher C3 level, which indicate lower disease activity, may contribute to physicians’ reluctance to prescribe HCQ for patients with stable SLE. This discrepancy can be clarified by the concept of “clinical inertia”, which refers to a failure of healthcare providers to initiate or intensify therapy when guidelines indicate doing so^22^. The presence of clinical inertia associated with long-term disease is also a barrier to appropriate therapeutic intervention in patients with rheumatoid and psoriatic arthritis^23,24^. However, HCQ should be recommended in essentially all patients, regardless of disease duration, because it can reduce relapse rates^4^. This is applicable even in situations where physicians insufficiently evaluate the necessity of prescribing HCQ in patients with a low PSL dose or stable serological markers. Additionally, it is noteworthy that C3, compared to dsDNA or clinical SLEDAI-2K, was associated with HCQ use. C3, which indicates alternative pathway activation, is more sensitive and specific to flares than C4, reflecting classical pathway activation and decreasing typically 2 months prior^25,26^. Conversely, dsDNA levels usually increase weeks to months before a flare (40–60%)^27–29^ and might decrease during a flare, indicating deposition as an immune complex^30^. Therefore, when a decrease in C3 level is observed on the day of patient visits, both physicians and patients will likely recognize other signs of worsening disease, possibly leading to more frequent recommendations of HCQ by physicians and patients’ acceptance. The practicality of C3, compared to the more cumbersome clinical SLEDAI-2K, and the unavailability of anti-dsDNA at the time of patient visits might also play a significant role. Furthermore, a distinct aspect of our findings should be noted: Attending physicians’ years since graduation, a factor that has been poorly studied, significantly influenced HCQ non-use. Extensive experience can lead to a deeper understanding of patient types and better clinical practice, sometimes resulting in deliberate nonconformity to guidelines^31,32^, possibly contributing to HCQ non-use. However, experienced physicians might be more resistant to adopting new therapies or provide lower quality care than fresher physicians^33,34^, which is another factor leading to the less frequent HCQ use.

The proportion of HCQ use at 50.2% in this study was higher than that in 18.3% of LUNA^15^. However, it was lower than the proportions in other countries, such as 76.7% in the Hopkins cohort in the United States^35^, 69.6% in the Birmingham cohort in the United Kingdom^36^, and 77.1% in the GLADEL cohort in Latin American countries^6^. The difference with LUNA may be affected by the implementation in subsequent years, which naturally leads to an increase in HCQ use because of accumulating evidence and guideline recommendations in Japan^3,37, 38^. Considering this study’s results and the LUNA database, the proportion of HCQ use in Japan is considered low compared to that of other countries. This can be attributed to the withdrawal of the domestic sale of chloroquine in 1974 in Japan because of a series of retinopathy cases, mainly due to additional indications approved in Japan for chronic nephritis^39^ and the consequent unavailability of HCQ until 2015. In fact, patients with SLE diagnosed before 2015 were less likely to use HCQ (85/202) than the others (45/55) in this study. Notably, the year of diagnosis could have also been a potential confounder because patients diagnosed before 2015, of course, have longer disease duration, probably take a lower PSL dose, and have had more opportunities to consult experienced physicians. However, the subgroup analysis for the patients diagnosed before 2015 demonstrated consistent results, supporting the association of the four factors and HCQ non-use.

This study has significant strengths, notably in its focus on uncovering the reasons behind the non-use of HCQ in Japan and the potential to contribute to resolving similar issues in other countries. In countries where HCQ is widely used, its usage proportion of approximately 70% may not be sufficiently high, particularly given that HCQ is recommended for all patients with SLE. The issue of adherence to HCQ is also well-known^40^. However, this study had some limitations. First, the data for the non-HCQ and HCQ groups were collected on different dates. Therefore, the percentage of HCQ use is not strictly precise. Although mycophenolate mofetil (MMF) and belimumab were approved in 2016 and 2017, respectively, which can make the two groups more heterogeneous, only few patients had data pertaining to variables before the approval of MMF or history of belimumab use. This also leads to inequalities in age and disease duration; however, the difference between the two groups was sufficiently large to be unaffected by the data collection date. Second, this was a single-center study, and since it was conducted in one hospital in Japan, it is unclear whether the findings can be applied to other communities with different healthcare systems or resources. Additionally, the number of physicians was low, and the decision to use HCQ depended on the discretion of each attending physician. Therefore, the trend may be different in other hospitals. Furthermore, the small number of rheumatologists was a possible confounding factor, but the presence or absence of a rheumatologist’s license was less likely to be associated with HCQ use, because physicians with > 10 years since graduation tended not to use HCQ regardless of their license. Third, unexplored factors such as patient anxiety or the history of retinopathy may relate to HCQ non-use, and these are the subject of future work. Finally, causality was not specific since this was an observational study.

In conclusion, the percentage of HCQ use in this study was lower than that in other countries, and longer disease duration, lower PSL dose, higher C3 level, and longer years since graduation of attending physicians were associated with HCQ non-use. However, there is a need to educate physicians and patients to enable HCQ use even with these factors.

### Supplementary Information

Supplementary Tables.

## Data Availability

The datasets generated during and/or analysed during the current study are available from the corresponding author on reasonable request.
