# Author Correction: An observational study to identify causative factors for not using hydroxychloroquine in systemic lupus erythematosus

**DOI:** 10.1038/s41598-024-64763-5

**Published:** 2024-06-14

**Authors:** Atsushi Manabe, Ryuichi Minoda Sada, Hirofumi Miyake, Hiroyuki Akebo, Yukio Tsugihashi, Kazuhiro Hatta

**Affiliations:** 1 Department of General Internal Medicine, Tenri Hospital, Tenri, Japan; 2 Department of Rheumatology and Clinical Immunology, Graduate School of Medicine, Kyoto University, Kyoto, Japan; 3 Department of Infection Control, Graduate School of Medicine, Osaka University, Suita, Japan; 4 Department of Transformative Protection to Infectious Disease, Graduate School of Medicine, Osaka University, 2-2 Yamadaoka, Suita, Osaka 565-0871 Japan; 5 Medical Home Care Centre, Tenri Hospital, Shirakawa Branch, Tenri, Japan

Correction to: *Scientific* *Reports* 10.1038/s41598-024-58463-3, published online 02 April 2024

The original version of this Article contained an error in the Materials and Methods section, under the subheading ‘Study design and settings’.

“This study protocol was approved by the Institutional Review Board of Tenri Hospital (Number 1212) and written informed consent was obtained from each patient as part of another study published by our facility^16^. This study was conducted in accordance with the principles of the Declaration of Helsinki.”

now reads:

“This study protocol was approved by the Institutional Review Board of Tenri Hospital (Number 1212) and was conducted in accordance with the tenets of the Declaration of Helsinki. An opt-out method was used to provide explanations to the patients and obtain consent.”

The original Article has been corrected.

