# Supplementary material for: An observational study to identify causative factors for not using hydroxychloroquine in systemic lupus erythematosus
Source: Sci Rep. 2024 Apr 2;14:7750. doi: 10.1038/s41598-024-58463-3 (PMC10987587; doi:10.1038/s41598-024-58463-3)
Supplement: Supplementary file 1 — Supplementary Tables. [file 41598_2024_58463_MOESM1_ESM.pdf]

An observational study to identify causative factors for not using hydroxychloroquine in systemic lupus erythematosus

Atsushi Manabe[1,2], Ryuichi Minoda Sada[1,3,4*], Hirofumi Miyake[1], Hiroyuki Akebo[1], Yukio Tsugihashi[5], and Kazuhiro Hatta[1]

[1] Department of General Internal Medicine, Tenri Hospital, Tenri, Japan

[2] Department of Rheumatology and Clinical Immunology, Graduate School of Medicine, Kyoto University, Kyoto, Japan

[3] Department of Infection Control, Graduate School of Medicine, Osaka University, Suita, Japan

[4] Department of Transformative Protection to Infectious Disease, Graduate School of Medicine, Osaka University, Suita, Japan

[5] Medical Home Care Centre, Tenri Hospital Shirakawa Branch, Tenri, Japan

***Corresponding author**

Ryuichi Minoda Sada, MD: Department of Transformative Protection to Infectious Disease, Graduate School of Medicine, Osaka University, 2-2 Yamadaoka, Suita, Osaka, 565-0871, Japan. Tel: +81-6-6879-5111. Email: sada0@cider.osaka-u.ac.jp

Supplementary Table S1. Univariate analysis for factors associated with non-use of hydroxychloroquine in patients with systemic lupus erythematosus

Variable	non-HCQ group (n=132)	HCQ group (n=133)	P-value
Sex (female), n/N (%)	121/132 (91.7%)	120/133 (90.2%)	0.83
Age (years)	57 (46–69)	45 (35–59)	<0.001
Disease duration (years)	20 (12–28)	5 (2–11)	<0.001
Clinical manifestations, n/N (%)			
Malar rash	67/132 (50.8%)	59/133 (44.4%)	0.33
Discoid rash	42/132 (31.8%)	33/133 (24.8%)	0.22
Photosensitivity	56/132 (42.4%)	51/133 (38.3%)	0.53
Oral ulcers	23/132 (17.4%)	15/133 (11.3%)	0.16
Arthritis	78/132 (59.1%)	77/133 (57.9%)	0.90
Serositis	24/132 (18.2%)	35/133 (26.3%)	0.14
Renal disorder	51/132 (38.6%)	43/133 (32.3%)	0.31
Neurological disorder	17/132 (12.9%)	12/133 (9.0%)	0.33
Hematologic disorder	116/132 (87.8%)	110/133 (82.7%)	0.30
Clinical SLEDAI-2K	2 (0–4)	4 (2–6)	<0.001
PSL (mg/day)	5 (2–6.3)	7 (4–15)	<0.001
Immunosuppressive drugs, n/N (%)	80/132 (60.6%)	79/133 (59.4%)	0.90
AZA, n/N (%)	8/132 (6.1)	7/133 (5.3)	0.80
TAC, n/N (%)	14/132 (10.6)	14/133 (10.5)	1.0
CyA, n/N (%)	17/132 (12.9)	15/133 (11.3)	0.71
MMF, n/N (%)	9/132 (6.8)	10/133 (7.5)	1.0
MTX, n/N (%)	2/132 (1.5)	5/133 (3.8)	0.45
MZR, n/N (%)	6/132 (4.5)	7/133 (5.3)	1.0
BLM, n/N (%)	1/132 (0.8)	0/133 (0)	0.50
eGFR (mL/min/1.73 m^2)	72.3 (55.5–83.6)	83.5 (68.5–100.7)	<0.001
AST (IU/L)	21 (17–28)	19 (16–27)	0.08
C3 (mg/dL)	85.0 (72.8–100.3)	75.0 (58.0–88.0)	<0.001
Anti-dsDNA	10.0 (4.0–23.0)	16.0 (6.0–41.0)	0.02
Attending physicians' years since graduation	8 (6–27)	37 (27–37)	<0.001

Data presented as median (interquartile range) of patients unless otherwise indicated. In the analysis of disease duration, clinical SLEDAI, eGFR and AST, C3, and Anti-dsDNA, the non-HCQ and HCQ groups consisted of 127 and 130, 132 and 132, 131 and 133, 128 and 133, and 122 and 119 patients, respectively. Abbreviations: HCQ, hydroxychloroquine; Clinical SLEDAI-2K, clinical systemic lupus erythematosus disease activity index 2000; PSL, prednisolone; eGFR, estimated glomerular filtration rate; AST, aspartate aminotransferase; AZA, azathioprine; TAC, tacrolimus; CyA, cyclosporine; MMF, mycophenolate mofetil; MTX, methotrexate; MZR, mizoribine; BLM, belimumab.

Supplementary Table S2. Multivariate analysis by logistic regression model for factors associated with non-use of hydroxychloroquine in patients with systemic lupus erythematosus (the categorical variables were changed to continuous variables)

Variable	Odds ratio (95% CI)	P-value
Female	0.72 (0.19–2.70)	0.62
Age, per year	1.00 (0.98–1.03)	0.81
Disease duration, per year	1.07 (1.03–1.11)	<0.001
Clinical SLEDAI-2K	0.93 (0.82–1.05)	0.22
PSL (mg/day)	0.86 (0.78–0.94)	0.001
eGFR (mL/min/1.73 m^2)	0.99 (0.97–1.00)	0.10
C3 (mg/dL)	1.02 (1.00–1.03)	0.046
Anti-dsDNA (IU/mL)	1.00 (1.00–1.00)	0.81
Attending physicians' years since graduation	1.06 (1.03–1.09)	<0.001

The logistic regression model analyzed non-use of hydroxychloroquine as the dependent variable against column variables as independent variables, including participants with no missing values (n=233). Abbreviations: CI, confidence interval; Clinical SLEDAI-2K, clinical systemic lupus erythematosus disease activity index 2000; PSL, prednisolone; eGFR, glomerular filtration rate.

Supplementary Table S3. Univariate analysis for factors associated with non-use of hydroxychloroquine in patients with systemic lupus erythematosus (the subgroup analysis for the patients diagnosed before 2015)

Variable	non-HCQ group (n=132)	HCQ group (n=133)	P-value
Sex (female), n/N (%)	107/117 (91.5%)	77/85 (90.6%)	1.0
Age (years)	57 (47–69)	45 (35–57)	<0.001
Disease duration (years)	21 (14–29)	9 (5–17)	<0.001
Clinical manifestations, n/N (%)			
Malar rash	61/117 (52.1%)	47/85 (55.3%)	0.67
Discoid rash	42/117 (35.9%)	27/85 (31.8%)	0.55
Photosensitivity	54/117 (46.2%)	36/85 (42.4%)	0.67
Oral ulcers	20/117 (17.1%)	14/85 (16.5%)	1.0
Arthritis	70/117 (59.8%)	52/85 (61.2%)	0.88
Serositis	20/117 (17.1%)	18/85 (21.2%)	0.47
Renal disorder	44/117 (37.6%)	27/85 (21.2%)	0.46
Neurological disorder	16/117 (13.6%)	9/85 (10.6%)	0.67
Hematologic disorder	105/117 (89.7%)	71/85 (83.5%)	0.21
Clinical SLEDAI-2K = 0, n/N (%)	30/117 (25.6%)	7/85 (8.2%)	0.002
PSL ≤7.5 mg/day, n/N (%)	104/117 (88.9%)	49/85 (57.6%)	<0.001
Immunosuppressive drugs, n/N (%)	45/117 (38.5%)	37/85 (43.5%)	0.47
AZA, n/N (%)	7/117 (6.0)	6/85 (7.1)	0.78
TAC, n/N (%)	13/117 (11.1)	9/85 (10.6)	1.0
CyA, n/N (%)	16/117 (13.7)	9/85 (10.6)	0.67
MMF, n/N (%)	7/117 (6.0)	3/85 (3.5)	0.52
MTX, n/N (%)	1/117 (0.9)	4/85 (4.7)	0.16
MZR, n/N (%)	4/117 (3.4)	7/85 (8.2)	0.21
BLM, n/N (%)	1/117 (0.9)	0/85 (0)	1.0
eGFR <60 mL/min/1.73 m^2, n/N (%)	34/117 (29.1%)	12/85 (14.1%)	0.02
AST >30 IU/L	21/117 (17.9%)	14/85 (16.5%)	0.85
C3 ≥73 mg/dL, n/N (%)	85/114 (74.6%)	43/85 (50.6%)	<0.001
Anti-dsDNA ≤12 IU/mL, n/N (%)	69/108 (63.9%)	34/77 (44.2%)	0.01
Attending physicians with >10 years since graduation, n/N (%)	98/117 (83.8%)	44/85 (51.7%)	<0.001

Data presented as median (interquartile range) of patients unless otherwise indicated. In columns related to C3 and dsDNA, the cut-off values represent the lower and upper limits of normal, respectively. Abbreviations: HCQ, hydroxychloroquine; Clinical SLEDAI-2K, clinical systemic lupus erythematosus disease activity index 2000; PSL, prednisolone; eGFR, estimated glomerular filtration rate; AST, aspartate aminotransferase; AZA, azathioprine; TAC, tacrolimus; CyA, cyclosporine; MMF, mycophenolate mofetil; MTX, methotrexate; MZR, mizoribine; BLM, belimumab.

Supplementary Table S4. Multivariate analysis by logistic regression model for factors associated with non-use of hydroxychloroquine in patients with systemic lupus erythematosus (the subgroup analysis for the patients diagnosed before 2015)

**Variable**	**Odds ratio (95% CI)**	**P-value**
Female	0.48 (0.10–2.33)	0.36
Age, per year	1.01 (0.98–1.04)	0.44
Disease duration, per year	1.07 (1.02–1.11)	0.004
Clinical SLEDAI-2K = 0	1.91 (0.49–7.37)	0.35
PSL ≤7.5 mg/day	5.02 (2.01–12.58)	0.001
eGFR <60 mL/min/1.73 m^2	1.92 (0.68–5.37)	0.22
C3 ≥73 mg/dL	2.19 (0.96–5.00)	0.06
Anti-dsDNA ≤12 IU/mL	1.35 (0.61–3.00)	0.47
Attending physicians with >10 years since graduation	3.77 (1.59–8.98)	0.003

The logistic regression model analyzed non-use of hydroxychloroquine as the dependent variable against column variables as independent variables, including participants with no missing values (n=233). In columns related to C3 and dsDNA, the cut-off values represent the lower and upper limits of normal, respectively. Abbreviations: CI, confidence interval; Clinical SLEDAI-2K, clinical systemic lupus erythematosus disease activity index 2000; PSL, prednisolone; eGFR, glomerular filtration rate.
